# *CSN1S1*, *CSN3* and *LPL*: Three Validated Gene Polymorphisms Useful for More Sustainable Dairy Production in the Mediterranean River Buffalo

**DOI:** 10.3390/ani14101414

**Published:** 2024-05-09

**Authors:** Alfredo Pauciullo, Giustino Gaspa, Yi Zhang, Qingyou Liu, Gianfranco Cosenza

**Affiliations:** 1 Department of Agricultural, Forest and Food Sciences, University of Turin, 10095 Grugliasco, Italy; 2 College of Animal Science and Technology, China Agricultural University, Beijing 100193, China; 3 School of Life Science and Engineering, Foshan University, Foshan 528225, China; 4 Department of Agriculture, University of Naples Federico II, 80055 Portici, Italy

**Keywords:** Mediterranean river buffalo, *CSN1S1*, *CSN3*, *LPL*, *SCD*, milk traits, validation study

## Abstract

**Simple Summary:**

Confirmation studies for SNPs associated with milk traits, identified through genome-wide or classic association approaches, are very rare in dairy animals and, to our knowledge, have not been carried out in river buffaloes. In this species, the candidate gene approach remains the most commonly applied method for identifying markers for selective breeding. In this study, we validated and confirmed the association of three SNPs in key genes (*CSN1S1*, *CSN3* and *LPL*) with milk yield, protein and fat. Our data represent a very important indication for the preselection of young bulls destined for breeding programs with a view to achieving more sustainable dairy production.

**Abstract:**

The search for DNA polymorphisms useful for the genetic improvement of dairy farm animals has spanned more than 40 years, yielding relevant findings in cattle for milk traits, where the best combination of alleles for dairy processing has been found in casein genes and in *DGAT1*. Nowadays, similar results have not yet been reached in river buffaloes, despite the availability of advanced genomic technologies and accurate phenotype records. The aim of the present study was to investigate and validate the effect of four single nucleotide polymorphisms (SNP) in the *CSN1S1*, *CSN3*, *SCD* and *LPL* genes on seven milk traits in a larger buffalo population. These SNPs have previously been reported to be associated with, or affect, dairy traits in smaller populations often belonging to one farm. A total of 800 buffaloes were genotyped. The following traits were individually recorded, monthly, throughout each whole lactation period from 2010 to 2021: daily milk yield (dMY, kg), protein yield (dPY, kg) and fat yield (dFY, kg), fat and protein contents (dFP, % and dPP, %), somatic cell count (SCC, 10^3^ cell/mL) and urea (mg/dL). A total of 15,742 individual milk test day records (2496 lactations) were available for 680 buffalo cows, with 3.6 ± 1.7 parities (from 1 to 13) and an average of 6.1 ± 1.2 test day records per lactation. Three out four SNPs in the *CSN1S1*, *CSN3* and *LPL* genes were associated with at least one of analyzed traits. In particular, the *CSN1S1* (AJ005430:c.578C>T) gave favorable associations with all yield traits (dMY, *p* = 0.022; dPY, *p* = 0.014; dFY, *p* = 0.029) and somatic cell score (SCS, *p* = 0.032). The *CSN3* (HQ677596: c.536C>T) was positively associated with SCS (*p* = 0.005) and milk urea (*p* = 0.04). Favorable effects on daily milk yield (dMY, *p* = 0.028), fat (dFP, *p* = 0.027) and protein (dPP, *p* = 0.050) percentages were observed for the *LPL*. Conversely, the *SCD* did not show any association with milk traits. This is the first example of a confirmation study carried out in the Mediterranean river buffalo for genes of economic interest in the dairy field, and it represents a very important indication for the preselection of young bulls destined for breeding programs aimed at more sustainable dairy production.

## 1. Introduction

The domestic water buffalo (*Bubalus bubalis*) is a tropical animal known for its remarkable ability to adapt to the environment and its efficient use of feed in conditions of forage shortage. The species originated in Southeast Asia, where 97% of the world’s buffalo population is still reared [1], and then spread westward, arriving in Syria, Egypt and then Western Europe [2]. Therefore, these animals are of major economic and cultural importance for many populations globally, providing milk, meat and draft power. Two buffalo sub-types exist: the swamp type (2n = 48), found exclusively in its native Asian continent, and the river type (2n = 50), which is more widely distributed across other continents. These buffalo sub-types differentiate for karyological, morphological and behavioral characteristics [3,4,5].

Italy is the European country with the greatest number of buffaloes raised. In recent years, the Italian buffalo population has increased from about 12,500 head in the 1950s to over 400,000 in 2019 [1], which represent about 85% of the entire European population. Such remarkable growth has been driven by the exploitation of buffalo milk and the national and international increase in the “Mozzarella di Bufala Campana PDO” demand. Recent data show a significant growth of the whole supply chain, with a turnover estimated at EUR 500 million, involving more than 20,000 operators and observing a 5% annual increase in exports (www.ismea.it, accessed on 6 May 2024). Despite this, domestic buffalo have received less attention and economic investment compared to other ruminants, suggesting significant potential for further improvement in this species.

The achievement of high production levels and efficiency in buffalo farming requires the optimization of numerous factors and processes, including genetic improvement. In this respect, although new knowledge has been acquired [6,7], a high contiguity assembly of the reference genome has been published [8] and the first SNP array specifically designed for buffaloes has become available [9], the use of genomic data remains limited. Therefore, nowadays, the estimation of genomic breeding values and the application of genomic selection in domestic buffalo are significantly delayed, as recently highlighted by Cesarani et al. [10]. In addition, genome-wide association studies (GWAS) in buffalo using the medium density 90K SNP array often identify candidate variants in intergenic regions near many potential genes of interest [11,12,13]. However, these findings frequently lack subsequent confirmation studies. For this reason, the candidate-gene approach is still today a valid method for the identification of genetic associations with milk production traits. At the same time, this approach provides valuable information for breeders’ associations, which, in the last decade, have promoted the selection of buffalo sires with favorable genotypes for milk traits (https://www.risbufala.it/?page_id=58841, accessed on 6 May 2024).

Milk yield [14,15,16,17,18], total protein and caseins [19,20,21,22,23], fat content, fat percentage and fatty acid composition [18,20,24,25,26,27,28,29,30], milking time [14], etc., are among the most studied traits and are of great interest to breeders’ associations due to their direct link to cheese yield and economic profitability. Genetic variability and associations with dairy traits have been found for many genes of economic interest (*CSN1S1*, *CSN1S2*, *CSN3*, *SCD*, *LPL*, *OXT*, *OXTR*, etc.). However, many of these association studies are limited to a single buffalo farm, with a limited number of samples, or carried out using single gene variants, for instance, the association between the protein percentage and SNP AJ005430: c.578C>T in *CSN1S1* (αs-1 casein) [21], or the milk yield and SNP FM876222: g.133A>C in *SCD* (Stearoyl-CoA Desaturase) [15]. Therefore, the aim of this study was to extend the genotyping of the four most promising SNPs in four genes of interest for selection goals (*CSN1S1*, *CSN3*, *SCD*, *LPL*) in a larger population and to validate the genetic relationships with milk traits for breeding purposes.

## 2. Materials and Methods

### 2.1. Sampling and DNA Isolation

Individual blood collection was performed in compliance with Italian national laws and regulations by official veterinarians of ASL (Local Sanitary Unit of the Ministry of Health) during routine farm prophylaxis procedures.

Sample collection was carried out on a total of 800 Italian Mediterranean river buffaloes belonging to 8 dairy farms mainly located in Campania region (Southern Italy).

Genomic DNA was isolated using the procedure described by Goossens and Kan [31]. Concentrations and OD_260/280_ ratios were measured with the Nanodrop ND-2000 Spectrophotometer (Thermo Fisher Scientific Inc., Waltham, MA, USA).

### 2.2. Genotyping

Genotyping was accomplished using PCR-based methods. In particular, a duplex Artificially Created Restriction Site (ACRS)-PCR described by Pauciullo et al. [22] was performed for the AJ005430: c.578C>T at the *CSN1S1* (*αs1-CN*) and HQ677596: c.536C>T at the *CSN3* (*k-CN*). Additionally, Restriction Fragment Length Polymorphism (RFLP)-PCR, as described by Gu et al. [28] and Gu et al. [27], was used for genotyping the FM876222: g.133A>C at *SCD* (Stearoyl-CoA desaturase) and the AWWX01438720.1: g14229A>G at *LPL* (Lipoprotein lipase), respectively. PCR amplification was carried out using BioRad T100 thermocycler (BioRad, Hercules, CA, USA). The digestion products were analyzed directly via electrophoresis on a 2.5% agarose gel in 0.5× TBE buffer and stained with SYBR green nucleic acid stain (Lonza Rockland Inc., Rockland, ME, USA) (Figure S1).

### 2.3. Phenotypes Collection and Dataset Editing

The phenotypic data for milk yield and composition were obtained from the official recording program of the Italian Association of Breeders (AIA) and they were used for this study under a cooperation agreement. Data included daily milk yield (dMY, kg), protein yield (dPY, kg) and fat yield (dFY, kg), fat and protein content (dFP, % and dPP, %), somatic cell count (SCC, 10^3^ cell/mL) and urea (mg/dL), and they were individually recorded each month throughout the whole lactation from 2010 to 2021 (*n* = 16,457 records). Only animals with both complete genotypes for the 4 SNPs (MAF > 0.05, *n* = 762) and lactation records were retained as valid records in successive analysis. A total of 15,742 individual milk test day records (2496 lactations) were available for 680 buffalo cows with 3.6 ± 1.7 parity (from 1 to 13) and 6.1 ± 1.2 test day records per lactation on average (Table 1).

Further data editing was performed prior to studying the association between SNP genotypes and milk phenotypes. The steps included the following: (i) Removal of outliers. Data that were unsound (greater than ±3.5 standard deviations) or had null values for DIM > 10 days were excluded; (ii) Transformation of SCC to SCS. Somatic cell counts (SCC) were transformed into somatic cell scores according to Ali and Shook [32] to standardize observations into a more analytical useful metric; (iii) Inclusion criteria for lactation records. Only buffalo cows with lactations that included at least 5 records were retained. Following these rigorous data preprocessing steps, the final dataset included 645 buffaloes.

### 2.4. Statistical Analyses

Descriptive statistics were performed on both SNP and phenotypic data. Minor allele frequencies and Hardy–Weinberg equilibrium tests were computed for all 4 genes. Pairwise Pearson correlations among milk traits were also calculated.

The effects of SNP genotypes on milk traits were assessed through mixed-model analysis, implementing 2 different genetic models using PROC MIXED from SAS^®^ (2016 Cary, NC, USA): allelic and genotypic models. In both genetic models, the fixed effects of the contemporary group were included, along with other systematic sources of variation, as specified thereafter. The type 3 sum of squares of PROC MIXED was computed, and SNP effects were considered significant for *p*-values < 0.05.

### 2.5. Allelic Model

In the first genetic model for each of the 4 investigated polymorphisms, the phenotypic values for milk traits were regressed onto the number of B allele (0, 1 or 2) for A/A, A/B and B/B genotypes, respectively, (Table 2) according to an additive model. Moreover, the effect of dominance was assessed with a different parametrization for dominant (1) and recessive genotypes (0). The general model used both for additive and dominance parametrization was:

(1) $$y_{ijklmo}=\mu +\beta \times SNP+{YEAR}_{i}+{DIM}_{j}+{NL}_{k}+{SEA}_{l}+{htd}_{m}+{bcow}_{n}+e_{ijklmn}$$

where y is the test-day phenotypic values for each analysed milk trait, μ is the mean of phenotypic values, SNP is the covariate of allelic count and β the average substitution effect for the additive model (AM), or dominance effect for the dominance model (DM). Moreover, the fixed effect of YEAR of birth (11 levels), days in milk (DIM: 15 classes of 20 d each), parity (NL, six classes, from 1 to 6+) and season of birth (SEA, 2 classes: autumn–winter and spring–summer) were fitted. Random effects for combination of herd-test day (htd, 669 levels), buffalo cows (bcow, 645 levels) and residual were also included. Random effects were assumed independently and identically distributed.

### 2.6. Genotypic Model

The genotypic model was similar to model (1), with the main difference that the genotypes at the four loci were treated as cross-classified fixed effects instead of as covariates (3 genotypic classes for A/A, A/B, B/B) according to:

(2) $$y_{ijklmo}=\mu +{SNP}_{i}+{YEAR}_{j}+{DIM}_{k}+{DIM(SNP)}_{k(i)}+{NL}_{l}+{SNP(NL)}_{i(l)}+{SEA}_{m}+{htd}_{n}+{bcow}_{o}+e_{ijklmno}$$

where DIM(SNP) represents the nested effect of days in milk within SNP genotype and SNP(NL) represents the genotypes nested within the parity effect. The other terms are the same as those in the previous model. In this model, a type 3 sum of square F-test for fixed effects was performed, and the marginal means of different genotypes were separated at *p*-values < 0.05 in post-hoc comparisons, adjusting the *p*-values according to Tukey HSD (adjust = Tukey of PROC MIXED).

Finally, to estimate the proportion of variance explained by the genotypes, a simplified model was used:

(3) $$y_{ijklmo}=\mu +{Year}_{i}+{DIM}_{j}+{NL}_{k}+{Season}_{l}+{SNP}_{m}+{htd}_{n}+{bcow}_{o}+e_{ijklmno}$$

where SNP genotypes, htd and cow are treated as random effects and the proportions of variance explained by the SNP genotype ($r_{SNP}^{2}$), herd-test day ($r_{htd}^{2}$) and buffalo cows ($r_{bcow}^{2}$) were computed, respectively, as the ratio of the variance components for each polymorphism to the total variance.

$${\hat{\sigma }}^{2}={\hat{\sigma }}_{SNP}^{2}+{\hat{\sigma }}_{htd}^{2}+{\hat{\sigma }}_{bcow}^{2}+{\hat{\sigma }}_{e}^{2}:\;r_{SNP}^{2}={\hat{\sigma }}_{SNP}^{2}/{\hat{\sigma }}^{2},\;r_{htd}^{2}={\hat{\sigma }}_{htd}^{2}∕{\hat{\sigma }}^{2}\mathrm{and}\;r_{Bcow}^{2}={\hat{\sigma }}_{bcow}^{2}∕{\hat{\sigma }}^{2}.$$

## 3. Results and Discussion

In the present study, four SNPs (AJ005430:c.578C>T, HQ677596:c.536C>T, FM876222:g.133A>C and AWWX01438720.1:g14229A>G), each located in a gene of interest for selection goals (*CSN1S1*, *CSN3*, *SCD* and *LPL*, respectively) were genotyped in a population of 800 Mediterranean river buffaloes across eight farms (Figure S1). The selection of these specific SNPs was driven by the need to confirm their impact on milk traits, as identified in previous studies carried out on relatively small buffalo populations [15,21,23,27]. In addition, two of these SNPs (AJ005430:c.578C>T in the *CSN1S1* and HQ677596:c.536C>T in the *CSN3*) were recently included in the genotyping program for buffalo sire selection by one of the two Italian Mediterranean buffalo breeders’ associations (Research Innovation and Selection for the buffalo).

The four investigated SNPs largely segregate in the buffalo population under study (MAF > 0.21, Table 2), with a variability range of 0.16–0.55 across genes or herds. With few exceptions, the four polymorphisms were in HW equilibrium both within and across herds (Figure S2, Table S1). Overall, deviation from the HW equilibrium was partially expected for *SCD* (χ^2^ = 6.19), which had previously been investigated in two different populations with similar findings (χ^2^ = 6.92, [15]; χ^2^ = 7.96, [28]). *SCD* FM876222: g.133A>C was associated with milk yield, and the allele substitution effect was assessed in about −1 kg/d, with 12% of the total phenotypic variance explained by polymorphism [15]. This effect is larger than that evidenced for *DGAT1* on milk yield in dairy cattle [33]. Despite this, so far, no marker-assisted selection has been voluntarily applied in favour of allele A to increase buffalo milk production. Therefore, the HW deviation for *SCD*, with the frequency of allele A nearly reaching 80%, can be considered as the result of farmers’ directional selection for more productive animals.

Conversely, the deviation from the HW principle for *CSN1S1* (χ^2^ = 5.06) was unexpected, given the findings of previous studies [21,22]. However, since 2021, the Italian buffalo population has been under selective pressure for the SNP AJ005430:c.578C>T (https://www.risbufala.it/?page_id=58841, accessed on 6 May 2024). Therefore, the observed HW deviation could potentially be considered the result of an artificial selection sweep.

For six milk traits, the number buffaloes with valid records were 645, with an average DIM of 153 ± 93 days. However, 29 animals were excluded from urea analysis due to missing phenotype data. The number of test days and lactation records varied slightly for the milk traits (from 20 to 22 records per animal on average). The average daily milk yield and composition, along with their pairwise phenotypic correlations (Table 3), are consistent with previous reports [10,14,15,19,34,35] and with the official average milk yield (8.70 ± 2.58 kg/d) reported for standard lactations (until 270 DIM) in 2022 [36]. Milk urea, which is important for its role in nitrogen metabolism, shows a weak correlation (<0.10) with all traits. Indeed, milk urea correlated positively with protein yield and negatively with fat content (Table 3).

This result is among the first indications of a correlation between milk urea and other milk parameters in buffaloes, as few studies are available in this species. Instead, more information is available in dairy cows, where more conflicting data have been reported. In general, a low negative genetic correlation has been found between milk urea and milk yield [37,38], but in New Zealand dairy cattle, the correlation between these two traits was reported as moderately positive [39,40]. Differences between diet formulations are considered as important factors that may cause genetic × environmental interactions that could explain such differences [37]. This could be also the case for the buffalo, whose genetic background, energy requirement and diet differ from those of dairy cattle.

With few exceptions (dFP and SCS in respect of birth season), all the fixed effects were highly significant (Table S2). Additive and dominance effects are reported in Table 4. In the allelic models, *LPL* showed a significant negative substitution effect on dMY when increasing the number of G alleles (*p* < 0.05) and a positive effect on fat and protein content of milk (dFP and dPP *p* < 0.05).

Considering that lipoprotein lipase (LPL) facilitates the hydrolysis of triglycerides transported via chylomicrons and very low-density lipoproteins, serving as a pivotal stage in the transportation of free fatty acids to mammary gland and adipose tissues, through its regulation of fatty-acid delivery to the mammary gland, *LPL* could influence the fat content of milk.

Our results are also consistent with recent findings in the Italian buffalo population. In fact, allele G showed a significant over-expression in homozygosity (~2.5-fold higher) compared with other genotypes and was associated with milk PUFA content [27]. Conversely, allele A showed higher values for milk yield in homozygosity, although the estimated difference from the other two genotypes only approached the level of significance (*p* = 0.07) [27]. Associations of *LPL* with milk fat traits and dMY have also been found in other species [41,42,43,44]. So far, no associations between *LPL* and milk proteins have been reported for buffaloes; however, a significant association was recently found in Czech dairy goats for this trait with the SNP *LPL* g.185G>T [42].

The investigated polymorphism at *CSN1S1* exhibited positive additive effects on dMY, dFY, dPY and SCS at increasing dose of T alleles (Table 4), whereas no significant effects of *CSN3* polymorphism was exerted on proteins (dPY and dPP) and other milk traits (dMY, dFY and urea), except for a higher SCS observed with an increasing number of T alleles (Table 4). Overall, this result confirms and reinforces the importance of the *αs1-CN* encoding gene in determining buffalo milk characteristics, with some important differences compared to the former study by Cosenza et al. [21]. The first difference is the higher number of dairy traits associated with the same SNP in the present study, although the protein percentage showed only a tendency in the genotypic model (*p* < 0.09), compared to being associated (*p* < 0.04) by Cosenza et al. [21]. However, the present dataset is more robust (2500 lactations, 8 farms, nearly 650 buffaloes) compared to the former study, which was numerically much smaller (500 lactations, 1 farm, 175 buffaloes). This difference also had other implications. Most notably, the allele substitution effect (cytosine into thymine) changed from the −0.014 observed by Cosenza et al. [21] to 0.011 in the present study. Differences of substitution effects across populations are possible and are influenced by several factors, such as the extent of variances (additive, dominance and additive by additive), the genetic distance of the populations and their heterozygosity [45]. The contribution of AJ005430:c.578C>T to the total phenotypic variance found by Cosenza et al. [21] was quite low (r^2^_αs1_ = 0.003) compared to the present study (r^2^_αs1_ = 0.100). Considering that Cosenza et al. [21] also found a large dominance effect (–0.028 ± 0.019), then altogether these data may explain, at least partially, the different results between the two studies.

The approached association (*p* < 0.06) of *CSN3* (κ-CN) in the genotypic model represents a further confirmation of the importance of this *locus* for milk traits. The HQ677596:c.536C>T alleles X1 (p.Ile^135^) and X2 (Thr^135^) are known to play a fundamental role in buffalo-milk processing, especially in combination with the variants AJ005430:c.578C>T, alleles B (p.Ser^178^) and A (Leu^178^) at *CSN1S1* [19,23]. In this context, the combined genotypes AA-X1X2 have shown better curd performance, with shorter rennet coagulation times, faster curd-firming time and greater curd firmness [19]. Conversely, the combination of alleles CSN1S1*B and CSN3*X1 resulted in higher curd yield [23]. Surprisingly, an association was also observed between both casein genes (*CSN1S1* and *CSN3*) and SCS. The allelic and genotypic models converged in identifying the polymorphism at *CSN1S1* gene for both additive (*p* < 0.05) and dominance (*p* < 0.05) effects on SCS. The average values for C/T and T/T buffalo genotypes did not differ significantly in the log-transformed somatic cell count at *p* < 0.05 (3.28 and 3.25), whereas the average for C/C genotypes was significantly lower than the former, thus configuring a degree of dominance of T over the C allele. Similarly, for *CSN3*, whose additive effect was significantly associated with SCS, a degree of dominance has also been observed for milk urea, where the heterozygous had significantly higher average values when compared to the opposite homozygous (Table 5).

Milk somatic cells consist of milk-secreting epithelial cells and immune cells. Regarding *CSN3*, it is known to have originated from the fibrinogen through a gene duplication event [46], and fibrinogen is one of the main mediators of the acute phase of inflammation [47]. Therefore, it is possible that the κ-casein has retained some of the functions of its ancestral gene and plays an active role as indicator of SCS and mastitis. Further support for this statement derives from the role that the κ-casein glycomacropeptide (GMP) plays in modulating the immune response, and its antibacterial and anti-inflammatory properties [48,49,50]. In addition, SNP rs43703017, located in *CSN3*, has recently been associated with an increase in SCS in domestic cattle [51]. Regarding *CSN1S1*, the association with SCS confirmed in buffalo underscores the significant impact of this gene as a promising candidate for selection to improve resistance against mastitis, as already suggested in dairy cows [52,53].

Regarding milk urea, no genes showed a significant substitution effect on this trait. The polymorphism in *SCD* does not appear to affect any of the investigated milk phenotypes for AM. Positive dominance effects are suggested (*p* < 0.05) for SCS (*CSN1S1*) and milk urea (*CSN3*).

The use of the genotypic model substantially confirmed the results of allelic model, with few differences in the significance levels for *LPL* (dMY, dPP), *αs1-CN* (dPY) *κ-CN* (dPP) that only approached the significant threshold (*p* < 0.10). However, with a good approximation, these results can still be considered suggestive of an SNP-phenotype association, as also confirmed by the proportion of variance explained by SNP effects for those trait-gene associations (from 0.2% to 0.4%) (Table 5). Indeed, the *LPL* polymorphism accounted for 0.3% and 0.2% of the total variability in dMY and dPP, respectively. The polymorphism at *CSN1S1* explained the 0.4% of total variance for dMY and dPY and SCS. Although the percentage of variance in absolute values was relatively small (0.1% to 0.7% cumulatively across traits), this is not unusual when the genetic association of single genes is analyzed.

It is worth noting that the random effects of buffalo cows and htd explained a large part of the variance. In general, it appears that variance accounted for buffaloes is larger for SCS and urea (25–57%) and smaller for milk yield and composition (8–14%). With an opposite trend, htd largely explains the intra-herd-test-day variability (26–37%) for dMY, dPY and dFY, but less so for milk contents, SCS and urea (7.5–14.5%). In this context, the different environmental and management conditions among the eight farms might not have allowed for better control of some sources of non-genetic variation. Therefore, the high level of variability observed in the present study may be attributed to the relevant impact of environmental factors.

**Figure 1:**
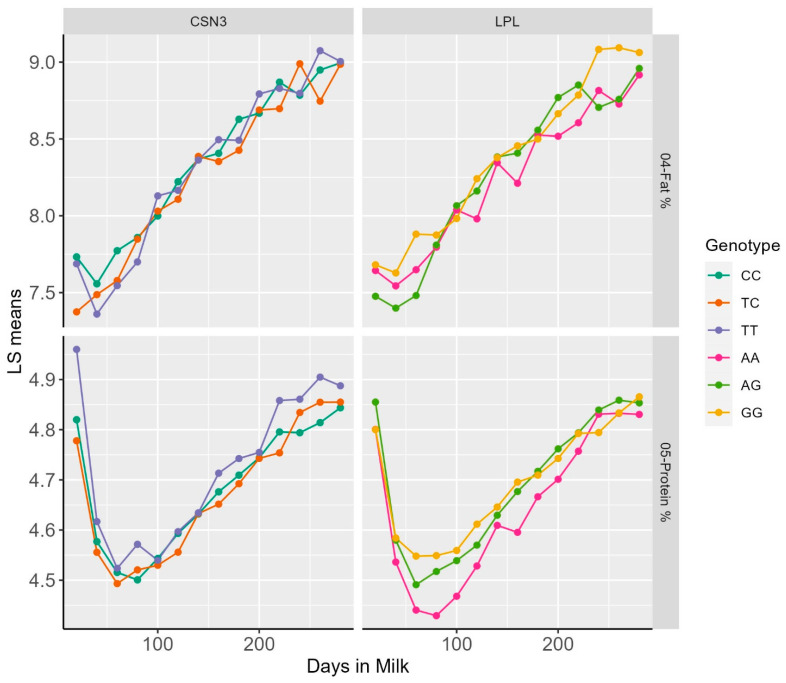
Plot of LS means of FP and PP over Days in milk within genotypic classes for the polymorphisms in the *LPL* and *CSN3* genes.

Representative examples of DIM classes and least square means for dPP and dFP for the *LPL*, as well as dPP and SCS for *CSN3,* are reported within the lactation patterns of different genotypes (Figure 1).

## 4. Conclusions

The genetic improvement of dairy traits is among the main goals of the Italian Mediterranean river buffalo association of breeders. In the present study, we have extended to a larger population the investigation on four polymorphisms that have been previously associated with dairy traits in a limited number of samples, often from a single farm. Three out of four SNPs—*CSN1S1*, *CSN3* and *LPL*—were associated with at least one of the analyzed traits (dMY, dPY, dFY, dPP, dFP, SCS and Urea) using both an allelic and a genotypic model. In particular, *CSN1S1* (AJ005430:c.578C>T) showed favorable associations with all yield traits (dMY, dPY, dFY) and SCS, whereas *CSN3* positively associated with SCS and Urea. Favorable effects on dMY, dFP and dPP were observed for *LPL*. Conversely, SCD did not show any association with milk traits. Overall, our results provide important indications for the preselection of young buffalo bulls for dairy traits, but they also highlight the importance of confirmation studies in larger populations to validate previous associations and enable more efficient setup of gene-assisted breeding programs.

## Figures and Tables

**Table 1:** Genotype frequency and database structure.

Genotype	Records	Buffaloes (%)	Lactations	NL_buffalo_ ± sd	TD_buffalo_ ± sd	TD_lact_ ± sd
*αs1-CN*						
CC	6527	280 (41.2)	1043	3.8 ± 1.7	23.3 ± 15.2	6.1 ± 1.6
CT	6638	291 (42.8)	1054	3.6 ± 1.7	22.8 ± 14.4	6.1 ± 1.5
TT	2257	109 (16.0)	399	3.3 ± 1.7	23.6 ± 12.9	6.4 ± 1.8
*κ-CN*						
CC	7229	314 (46.2)	1171	3.7 ± 1.7	23.0 ± 14.7	6.0 ± 1.5
CT	6294	282 (41.5)	991	3.7 ± 1.7	22.2 ± 14.5	6.3 ± 1.6
TT	2219	84 (12.3)	334	3.4 ± 1.7	26.4 ± 12.3	6.7 ± 1.7
*SCD*						
AA	9920	425 (62.5)	1570	3.7 ± 1.7	23.3 ± 14.6	6.2 ± 1.6
AC	4781	211 (31.0)	765	3.6 ± 1.7	22.7 ± 13.8	6.1 ± 1.6
CC	1041	44 (6.50)	161	3.7 ± 1.7	23.7 ± 16.0	6.1 ± 1.6
*LPL*						
AA	1943	94 (13.9)	303	3.1 ± 1.7	20.7 ± 12.9	6.3 ± 1.7
AG	7450	319 (46.9)	1190	3.5 ± 1.7	23.4 ± 14.3	6.1 ± 1.5
GG	6349	267 (39.2)	1003	3.9 ± 1.7	23.8 ± 15.1	6.2 ± 1.6
Total	15,742	680 (100)	2496	3.6 ± 1.7	23.2 ± 14.5	6.1 ± 1.2

NL = parity; TD = Test day.

**Table 2:** Allele Frequency (minor alleles are in boldface).

Gene	Product	SNP	Position (Nucleotide)	Alleles	Genotypes	MAF
*CSN1S1*	αs1-casein	AJ005430:c.578C>T	Exon 17 (89)	C/**T**	A/B	0.37
*CSN3*	κ-casein	HQ677596:c.536C>T	Exon 4 (377)	C/**T**	A/B	0.33
*SCD*	Stearoyl CoA Desaturase	FM876222:g.133A>C	Promoter (−461)	A/**C**	A/B	0.21
*LPL*	Lipoprotein Lipase	AWWX01438720.1:g14229A>G	Exon 1 (107)	**A**/G	A/B	0.37

**Table 3:** Descriptive statistics and pairwise Pearson correlations for milk traits after data editing procedure.

	Descriptive					Pearson Correlation					
Trait ^1^	Records (TD ± sd) ^2^	N Buffaloes ^3^	Mean ± sd	Min	Max	dFY	dPY	dFP	dPP	SCS	Urea
dMY (kg/d)	14,219 (22.5 ± 14.0)	645	8.81 ± 4.15	0.20	26.8	0.90	0.97	−0.21	−0.24	−0.18	0.08
dFY (kg/d)	14,222 (22.1 ± 13.5)	645	0.74 ± 0.35	0.02	3.27		0.90	0.18	−0.12	−0.16	0.06
dPY (kg/d)	14,303 (22.2 ± 13.6)	645	0.40 ± 0.19	0.01	1.27			−0.16	−0.06	−0.17	0.09
dFP (g/100 g)	14,222 (22.1 ± 13.5)	645	8.52 ± 1.68	3.52	15.42				0.31	0.04	−0.05
dPP (g/100 g)	14,306 (22.2 ± 13.6)	645	4.70 ± 0.42	3.02	6.85					0.06	0.03
SCS (log)	13,738 (22.1 ± 13.5)	645	3.18 ± 1.90	−3.64	10.86						0.04
Urea (mg/dL)	12,212 (19.8 ± 12.3)	616	37.16 ± 13.46	0.12	145.2						
DIM	14,519 (22.5 ± 14.0)	645	152.69 ± 92.67	5.00	679						

^1^ dMY = daily milk yield, dFY = daily fat yield, dPY = daily protein yield, dFP = daily fat percent, dPP = daily protein percent, DIM = days in milk. ^2^ Number of valid records for animals without missing genotype or phenotype (average Test-Day per buffalo). ^3^ Number of used genotypes for statistical analysis.

**Table 4:** Allele effects: additive (α) and dominance (d) components of genes for the 7 analyzed phenotypic traits. Asterisks indicate significant results for *p* values < 0.05 (*) and values < 0.01 (**).

		Additive				Dominance			
Trait ^1^	Gene	α	s.e.	*p*		d	s.e.	*p*	
dMY (kg/d)	*CSN1S1*	0.237	0.104	0.022	*	0.224	0.148	0.131	
	*CSN3*	0.078	0.106	0.463		−0.002	0.149	0.988	
	*SCD*	−0.106	0.120	0.374		0.087	0.159	0.585	
	*LPL*	−0.238	0.108	0.028	*	0.177	0.147	0.229	
dFY (kg/d)	*CSN1S1*	0.018	0.008	0.029	*	0.015	0.012	0.210	
	*CSN3*	0.005	0.009	0.595		−0.004	0.012	0.718	
	*SCD*	−0.012	0.010	0.213		0.008	0.013	0.512	
	*LPL*	−0.012	0.009	0.183		0.010	0.012	0.399	
dPY (kg/d)	*CSN1S1*	0.011	0.005	0.014	*	0.008	0.007	0.255	
	*CSN3*	0.005	0.005	0.300		−0.002	0.007	0.785	
	*SCD*	−0.005	0.005	0.317		0.005	0.007	0.503	
	*LPL*	−0.008	0.005	0.098		0.008	0.007	0.208	
dFP (g/100 g)	*CSN1S1*	0.003	0.033	0.937		−0.035	0.047	0.461	
	*CSN3*	−0.031	0.034	0.354		−0.074	0.047	0.115	
	*SCD*	−0.052	0.038	0.164		−0.003	0.050	0.953	
	*LPL*	0.076	0.035	0.027	*	−0.047	0.046	0.312	
dPP (g/100 g)	*CSN1S1*	0.011	0.010	0.260		−0.018	0.014	0.182	
	*CSN3*	0.012	0.010	0.212		−0.019	0.014	0.173	
	*SCD*	−0.005	0.011	0.639		0.007	0.015	0.648	
	*LPL*	0.020	0.010	0.050	*	0.007	0.014	0.631	
SCS (log(SCC/100) + 3)	*CSN1S1*	0.087	0.041	0.032	*	0.119	0.057	0.038	*
	*CSN3*	0.117	0.041	0.005	**	0.067	0.058	0.247	
	*SCD*	−0.081	0.046	0.080		−0.076	0.061	0.216	
	*LPL*	0.008	0.042	0.845		−0.017	0.057	0.770	
UREA (mg/dL)	*CSN1S1*	−0.172	0.262	0.511		0.317	0.367	0.388	
	*CSN3*	0.177	0.266	0.507		0.909	0.365	0.013	*
	*SCD*	0.208	0.293	0.477		0.362	0.390	0.353	
	*LPL*	−0.029	0.271	0.915		−0.191	0.361	0.596	

**^1^** dMY = daily milk yield, dFY = daily fat yield, dPY = daily protein yield, dFP = daily fat percent, dPP = daily protein percent.

**Table 5:** Least square means of genotypic class for *CSN1S1*, *CSN3*, *SCD* and *LPL* genes on milk traits and proportion of variance explained by SNP, buffaloes and herd-test day effects.

				Genotype ^3^					% Variance Explained by Random Effect		
Trait ^1^	Gene	A/B	Allelic ^2^	A/A	A/B	B/B	P ^4^		r^2^_SNP_	r^2^_bcow_	r^2^_htd_
dMY (kg/d)	*CSN1S1*	C/T	*	8.00 ^b^_(0.12)_	8.32 ^ab^_(0.14)_	8.47 ^a^_(0.20)_	0.04	*	0.4	8.6	37.1
	*CSN3*	C/T		8.15 _(0.13)_	8.20 _(0.14)_	8.39 _(0.22)_	0.60	ns	0.0	8.7	37.3
	*SCD*	A/C		8.21 _(0.12)_	8.22 _(0.15)_	7.90 _(0.30)_	0.57	ns	0.0	8.7	37.3
	*LPL*	A/G	*	8.46 _(0.21)_	8.29 _(0.13)_	8.01 _(0.14)_	0.08	†	0.3	8.7	37.1
dFY (kg/d)	*CSN1S1*	C/T	*	0.66 _(0.01)_	0.68 _(0.01)_	0.70 _(0.02)_	0.08	†	0.3	9.6	26.2
	*CSN3*	C/T		0.67 _(0.01)_	0.67 _(0.01)_	0.69 _(0.02)_	0.59	ns	0.0	9.6	26.2
	*SCD*	A/C		0.68 _(0.01)_	0.68 _(0.01)_	0.64 _(0.02)_	0.22	ns	0.0	9.6	26.2
	*LPL*	A/G		0.69 _(0.02)_	0.68 _(0.01)_	0.67 _(0.01)_	0.46	ns	0.0	9.6	26.2
dPY (kg/d)	*CSN1S1*	C/T	*	0.37 ^b^_(0.01)_	0.38 ^ab^_(0.01)_	0.40 ^a^_(0.01)_	0.03	*	0.4	10.0	33.8
	*CSN3*	C/T		0.38 _(0.01)_	0.38 _(0.01)_	0.39 _(0.01)_	0.32	ns	0.0	10.1	34.0
	*SCD*	A/C		0.38 _(0.01)_	0.38 _(0.01)_	0.36 _(0.01)_	0.39	ns	0.0	10.1	34.0
	*LPL*	A/G		0.39 _0(.01)_	0.39 _(0.01)_	0.37 _(0.01)_	0.21	ns	0.1	10.1	34.0
dFP (g/100 g)	*CSN1S1*	C/T		8.33 _(0.06)_	8.28 _(0.06)_	8.34 _(0.08)_	0.59	ns	0.0	13.6	8.8
	*CSN3*	C/T		8.34 _(0.06)_	8.26 _(0.06)_	8.32 _(0.08)_	0.29	ns	0.0	13.6	8.8
	*SCD*	A/C		8.33 _(0.06)_	8.31 _(0.07)_	8.15 _(0.10)_	0.19	ns	0.0	13.6	8.8
	*LPL*	A/G	*	8.24 ^ab^_(0.08)_	8.27 ^b^_(0.06)_	8.38 ^a^_(0.06)_	0.05	*	0.1	13.6	8.8
dPP (g/100 g)	*CSN1S1*	C/T		4.68 _(0.02)_	4.67 _(0.02)_	4.72 _(0.02)_	0.09	†	0.1	14.3	14.5
	*CSN3*	C/T		4.68 _(0.02)_	4.68 _(0.02)_	4.73_(0.02)_	0.06	†	0.2	14.3	14.5
	*SCD*	A/C		4.69 _(0.01)_	4.69 _(0.02)_	4.65 _(0.03)_	0.43	ns	0.0	14.3	14.6
	*LPL*	A/G	*	4.64 _(0.02)_	4.69 _(0.02)_	4.70 _(0.02)_	0.06	†	0.2	14.3	14.5
SCS (log(SCC/100) + 3)	*CSN1S1*	C/T	*	3.12 ^b^_(0.08)_	3.28 ^a^_(0.08)_	3.25 ^ab^_(0.10)_	0.04	*	0.2	25.6	11.7
	*CSN3*	C/T	*	3.13 ^b^_(0.02)_	3.26 ^ab^_(0.08)_	3.35 ^a^_(0.02)_	0.03	*	0.3	25.5	11.7
	*SCD*	A/C		3.24 _(0.07)_	3.16 _(0.08)_	3.07 _(0.13)_	0.22	ns	0.1	25.7	11.7
	*LPL*	A/G		3.20 _(0.10)_	3.19 _(0.07)_	3.22 _(0.08)_	0.91	ns	0.0	25.7	11.7
UREA (mg/dL)	*CSN1S1*	C/T		37.59 _(0.62)_	37.68 _(0.62)_	36.77 _(0.73)_	0.23	ns	0.0	57.1	7.6
	*CSN3*	C/T		37.24 ^b^_(0.62)_	38.04 ^a^_(0.62)_	36.80 ^b^_(0.76)_	0.04	*	0.1	57.1	7.5
	*SCD*	A/C		37.45 _(0.60)_	37.72 _(0.65)_	37.35 _(0.89)_	0.77	ns	0.0	57.1	7.6
	*LPL*	A/G		38.00 _(0.75)_	37.38 _(0.61)_	37.60 _(0.63)_	0.54	ns	0.0	57.1	7.6

^1^ dMY = daily milk yield, dFY = daily fat yield, dPY = daily protein yield, dFP = daily fat percent, dPP = daily protein percent. ^2^ The genotype marked with an asterisk * was also significantly associated in the allelic model, whereas the genotype indicated by a dagger † showed a tendency towards significance, with a *p*-value < 0.10. ^3^ Marginal means of different genotypes with letters are separated at *p*-values < 0.05 in post-hoc comparison adjusting *p*-values according to Tukey–Kramer (HSD). ^4^ *p*-values for type III sum of square F-test for fixed effects.

## Data Availability

The data presented in this study are available on request from the corresponding author.

## Supplementary Materials

The following supporting information can be downloaded at: https://www.mdpi.com/article/10.3390/ani14101414/s1, Figure S1: Genotyping by duplex ACRS-PCR (*CSN1S1* and *CSN3*) and PCR-RFLP (*LPL* and *SCD*) for the four investigated SNPs (*CSN1S1* = AJ005430:c.578C>T; *CSN3 =* HQ677596:c.536C>T; *LPL* = AWWX01438720.1:g14229A>G and *SCD* = FM876222:g.133A>C); Figure S2: Minor allele frequencies (MAF) detected in the eight investigated herds for the four SNPs (*CSN1S1* = AJ005430:c.578C>T; *CSN3* = HQ677596:c.536C>T; *SCD* = FM876222:g.133A>C; *LPL* = AWWX01438720.1:g14229A>G); Table S1: Within-herd genotypic frequency, observed and expected heterozygosity and *p*-values for Hardy–Weinberg tests for the four investigated SNPs; Table S2: *p*-values for fixed effect included in the statistical model for the seven analyzed traits.
