# Supplementary material for: CSN1S1, CSN3 and LPL: Three Validated Gene Polymorphisms Useful for More Sustainable Dairy Production in the Mediterranean River Buffalo
Source: Animals (Basel). 2024 May 9;14(10):1414. doi: 10.3390/ani14101414 (PMC11117199; doi:10.3390/ani14101414)
Supplement: Supplementary file 1 [file animals-14-01414-s001.zip › animals-2981061-supplementary.pdf]

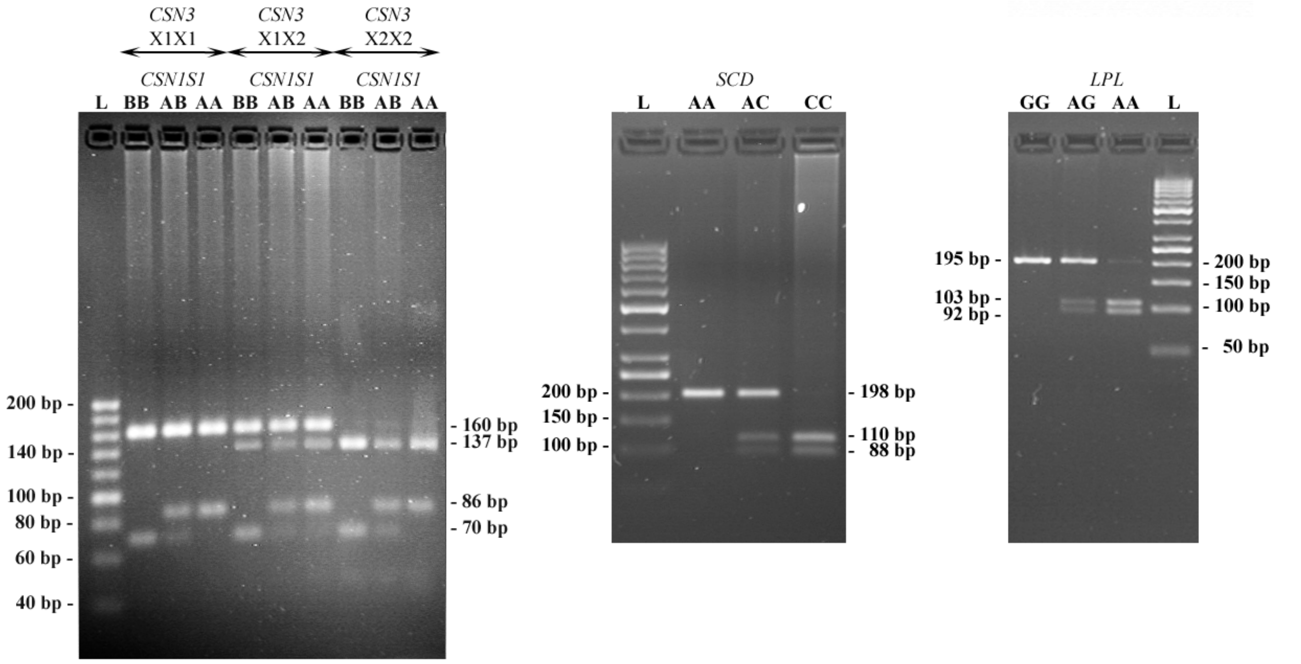

Figure S1. Genotyping by duplex ACRS-PCR (*CSN1S1* and *CSN3*) and PCR-RFLP (*LPL* and *SCD*) for the four investigated SNPs (*CSN1S1* = AJ005430:c.578C>T; *CSN3* = HQ677596:c.536C>T; *LPL* = AWWX01438720.1:g14229A>G and *SCD* = FM876222:g.133A>C).

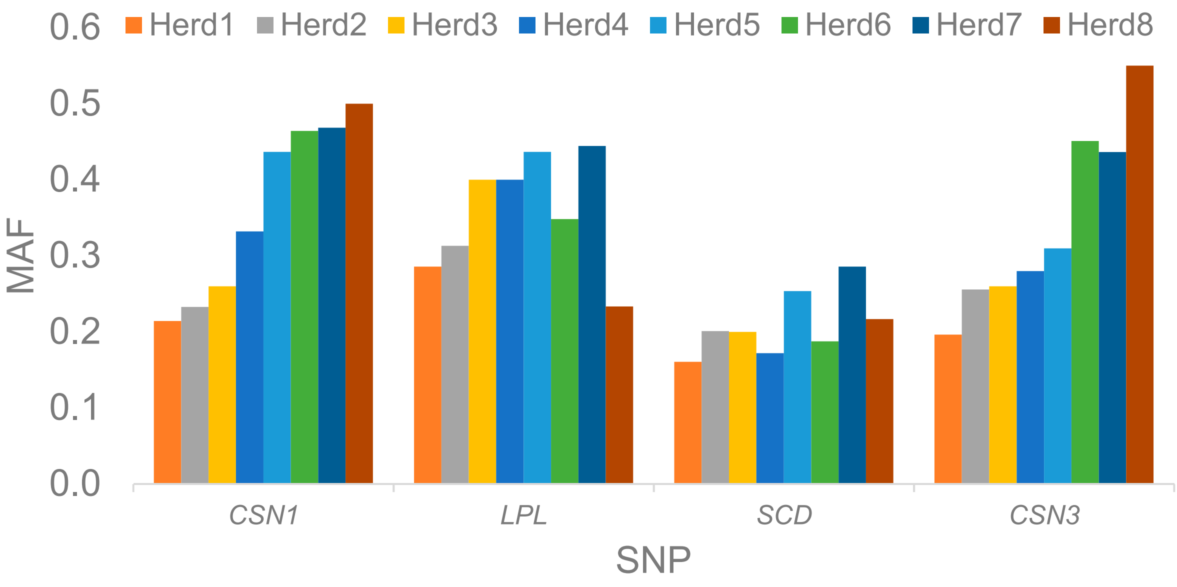

Figure S2. Minor allele frequencies (MAF) detected in the eight investigated herds for the four SNPs (*CSN1S1* = AJ005430:c.578C>T; *CSN3* = HQ677596:c.536C>T; *SCD* = FM876222:g.133A>C; *LPL* = AWWX01438720.1:g14229A>G).

Table S1. Within herd genotypic frequency, observed and expected heterozygosity and p-values for Hardy-Weinberg tests for the four investigated SNPs.

Herd	Gene[1]	A	B	Geno	O(het)	E(het)	P
1	*CSN1S1*	T	C	0/12/15	0.44	0.35	0.289
1	*CSN3*	T	C	0/11/16	0.41	0.32	0.549
1	*SCD*	C	A	1/5/21	0.19	0.23	0.358
1	*LPL*	A	G	1/14/12	0.52	0.42	0.367
2	*CSN1S1*	T	C	7/53/91	0.35	0.35	1.000
2	*CSN3*	T	C	6/63/82	0.42	0.37	0.193
2	*SCD*	C	A	12/37/102	0.25	0.32	**0.005**
2	*LPL*	A	G	10/70/71	0.46	0.42	0.244
3	*CSN1S1*	T	C	0/12/11	0.52	0.39	0.270
3	*CSN3*	T	C	0/12/11	0.52	0.39	0.270
3	*SCD*	C	A	01/07/15	0.30	0.31	1.000
3	*LPL*	A	G	3/12/8	0.52	0.48	1.000
4	*CSN1S1*	T	C	8/37/30	0.49	0.46	0.616
4	*CSN3*	T	C	3/37/35	0.49	0.41	0.096
4	*SCD*	C	A	4/20/51	0.27	0.30	0.267
4	*LPL*	A	G	16/29/30	0.39	0.48	0.095
5	*CSN1S1*	T	C	42/95/68	0.46	0.49	0.398
5	*CSN3*	T	C	27/73/105	0.36	0.43	**0.021**
5	*SCD*	C	A	10/84/111	0.41	0.38	0.274
5	*LPL*	A	G	36/107/62	0.52	0.49	0.477
6	*CSN1S1*	T	C	25/47/34	0.44	0.50	0.327
6	*CSN3*	T	C	24/46/36	0.43	0.49	0.238
6	*SCD*	C	A	5/31/70	0.29	0.31	0.533
6	*LPL*	A	G	14/45/47	0.42	0.45	0.524
7	*CSN1S1*	T	C	19/21/23	0.33	0.50	**0.011**
7	*CSN3*	T	C	15/25/23	0.40	0.49	0.129
7	*SCD*	C	A	8/20/35	0.32	0.41	0.117
7	*LPL*	A	G	12/32/19	0.51	0.49	1.000
8	*CSN1S1*	T	C	8/14/8	0.47	0.50	0.723
8	*CSN3*	C	T	6/15/9	0.50	0.50	1.000
8	*SCD*	C	A	3/7/20	0.23	0.34	0.101
8	*LPL*	A	G	2/10/18	0.33	0.36	0.632

[1]SNP is: *CSN1S1* = AJ005430:c.578C>T; *CSN3* = HQ677596:c.536C>T; *SCD* = FM876222:g.133A>C; *LPL* = AWWX01438720.1:g14229A>G.

Table S2. P-values for fixed effect included in the statistical model for the six analyzed traits.

Milk Trait[1]	Gene[2]	Year	Sea	NL	DIM	Dim(Genotype)	Genotype(NL)
	CSN1S1	<0.001	<0.001	<0.001	<0.001	0.044	0.003
	CSN3	<0.001	<0.001	<0.001	<0.001	0.600	<0.001
dMY	*SCD*	<0.001	<0.001	<0.001	<0.001	0.566	0.241
(kg/d)	*LPL*	<0.001	<0.001	<0.001	<0.001	0.081	0.463
	CSN1S1	<0.001	0.003	<0.001	<0.001	0.075	<0.001
	CSN3	<0.001	0.004	<0.001	<0.001	0.592	<0.001
dFY	*SCD*	<0.001	0.004	<0.001	<0.001	0.221	0.509
(kg/d)	*LPL*	<0.001	0.005	<0.001	<0.001	0.460	0.258
	CSN1S1	<0.001	<0.001	<0.001	<0.001	0.030	0.009
	CSN3	<0.001	<0.001	<0.001	<0.001	0.323	0.001
dPY	*SCD*	<0.001	<0.001	<0.001	<0.001	0.385	0.520
(kg/d)	*LPL*	<0.001	<0.001	<0.001	<0.001	0.206	0.778
	CSN1S1	<0.001	0.297	<0.001	<0.001	0.587	0.011
	CSN3	<0.001	0.353	<0.001	<0.001	0.286	0.196
dFP	*SCD*	<0.001	0.304	<0.001	<0.001	0.190	0.239
(g/100g)	*LPL*	<0.001	0.229	0.001	<0.001	0.051	0.010
	CSN1S1	<0.001	0.038	<0.001	<0.001	0.093	0.047
	CSN3	<0.001	0.039	<0.001	<0.001	0.063	0.094
dPP	*SCD*	<0.001	0.017	<0.001	<0.001	0.433	0.038
(g/100g)	*LPL*	<0.001	0.035	<0.001	<0.001	0.061	0.159
	CSN1S1	<0.001	0.130	<0.001	<0.001	0.038	0.000
	CSN3	<0.001	0.153	<0.001	<0.001	0.025	0.085
SCS	*SCD*	<0.001	0.133	<0.001	<0.001	0.222	0.001
(log(SCC/100)+3)	*LPL*	<0.001	0.109	<0.001	<0.001	0.909	0.100
	CSN1S1	<0.001	0.002	<0.001	<0.001	0.228	0.065
	CSN3	<0.001	0.003	<0.001	<0.001	0.039	0.326
UREA	*SCD*	<0.001	0.003	<0.001	<0.001	0.771	0.442
(mg/dL)	*LPL*	<0.001	0.004	<0.001	<0.001	0.538	0.304

[1] dMY=daily milk yield, dFY = daily fat yield, dPY = daily protein yield, dFP = daily fat percent, dPP = daily protein percent. [2]SNP is: *CSN1S1* = AJ005430:c.578C>T; *CSN3* = HQ677596:c.536C>T; *SCD* = FM876222:g.133A>C; *LPL* = AWWX01438720.1:g14229A>G.
